# Cytochrome P450 1 Genes in Birds: Evolutionary Relationships and Transcription Profiles in Chicken and Japanese Quail Embryos

**DOI:** 10.1371/journal.pone.0028257

**Published:** 2011-12-02

**Authors:** Maria E. Jönsson, Bruce R. Woodin, John J. Stegeman, Björn Brunström

**Affiliations:** 1 Department of Environmental Toxicology, Uppsala University, Uppsala, Sweden; 2 Biology Department, Woods Hole Oceanographic Institution, Woods Hole, Massachusetts, United States of America; Glasgow Caledonian University, United Kingdom

## Abstract

**Background:**

Cytochrome P450 1 (*CYP1*) genes are biomarkers for aryl hydrocarbon receptor (AHR) agonists and may be involved in some of their toxic effects. CYP1s other than the CYP1As are poorly studied in birds. Here we characterize avian *CYP1B* and *CYP1C* genes and the expression of the identified *CYP1* genes and *AHR1*, comparing basal and induced levels in chicken and quail embryos.

**Methodology/Principal Findings:**

We cloned cDNAs of chicken *CYP1C1* and quail *CYP1B1* and *AHR1*. *CYP1C*s occur in several bird genomes, but we found no *CYP1C* gene in quail. The *CYP1C* genomic region is highly conserved among vertebrates. This region also shares some synteny with the *CYP1B* region, consistent with *CYP1B* and *CYP1C* genes deriving from duplication of a common ancestor gene. Real-time RT-PCR analyses revealed similar tissue distribution patterns for *CYP1A4*, *CYP1A5*, *CYP1B1*, and *AHR1* mRNA in chicken and quail embryos, with the highest basal expression of the *CYP1A*s in liver, and of *CYP1B1* in eye, brain, and heart. Chicken *CYP1C1* mRNA levels were appreciable in eye and heart but relatively low in other organs. Basal transcript levels of the *CYP1A*s were higher in quail than in chicken, while *CYP1B1* levels were similar in the two species. 3,3′,4,5,5′-Pentachlorobiphenyl induced all *CYP1s* in chicken; in quail a 1000-fold higher dose induced the *CYP1As*, but not *CYP1B1*.

**Conclusions/Significance:**

The apparent absence of *CYP1C1* in quail, and weak expression and induction of *CYP1C1* in chicken suggest that *CYP1C*s have diminishing roles in tetrapods; similar tissue expression suggests that such roles may be met by *CYP1B1*. Tissue distribution of *CYP1B* and *CYP1C* transcripts in birds resembles that previously found in zebrafish, suggesting that these genes serve similar functions in diverse vertebrates. Determining CYP1 catalytic functions in different species should indicate the evolving roles of these duplicated genes in physiological and toxicological processes.

## Introduction

Members of the cytochrome P450 (CYP) superfamily of enzymes are present in most organisms, including bacteria, archaea, plants, fungi, and animals. They catalyze oxidative metabolism of various endogenous and exogenous compounds. Endogenous substrates include eicosanoids, cholesterol, bile acids, steroids, biogenic amines, vitamin D3, and retinoids [1], [2]. Enzymes in the CYP1, CYP2, CYP3, and CYP4 families also metabolize exogenous compounds, such as plant or fungal secondary metabolites, environmental pollutants, and pharmaceuticals [3], [4]. The CYP1 enzymes have been studied extensively because they can generate reactive and sometimes carcinogenic metabolites from environmental pollutants (e.g., polycyclic aromatic hydrocarbons, PAHs), but the interest in their endogenous functions is growing [e.g., [5]].

Genes in four *CYP1* subfamilies - *CYP1A*, *CYP1B*, *CYP1C*, and *CYP1D* - are expressed in fish and amphibians, while mammalian species express *CYP1A*, *CYP1B*, and in some cases *CYP1D* genes [6], [7], [8], [9], [10], [11], [12]. In fish and the frog *Xenopus tropicalis*, expression of *CYP1A*, *CYP1B* and *CYP1C* genes is induced by exposure to agonists of the aryl hydrocarbon receptor (AHR), among the most potent being 2,3,7,8-tetrachlorodibenzo-*p*-dioxin (TCDD) and 3,3′,4,4′,5-pentachlorobiphenyl (PCB126); *CYP1D* genes do not seem to be inducible by AHR agonists [6], [7], [10], [13].

Avian species vary substantially in sensitivity to embryo toxicity of halogenated aromatic hydrocarbons that activate the AHR [14], [15]. Chicken embryos are particularly sensitive to these compounds and the effects of exposure *in ovo* include reduced hatchability, developmental abnormalities, and induction of CYP1A-catalyzed enzyme activity [16], [17], [18]. Japanese quail embryos are considerably less sensitive than chicken embryos to TCDD and PCB126, both in terms of embryo toxicity and ethoxyresorufin *O*-deethylase (EROD) induction [19], [20], [21]. The difference in sensitivity has been attributed to variations in a few amino acid residues in the AHR [15], [22].

Birds have two *CYP1A* genes, *CYP1A4* and *CYP1A5*, which are orthologous to mammalian *CYP1A1* and *CYP1A2* [23] and which are inducible by AHR agonists [9], [24]. At least some bird species also express *CYP1B1*; the constitutive localization of *CYP1B1* mRNA has been determined in embryonic chicken (*Gallus gallus*) and quail (*Coturnix coturnix japonica*) [8], but the inducibility of *CYP1B1* in birds has not been reported. A *CYP1C* gene was identified recently in the chicken genome [25], and *CYP1C*s also appear in the Ensembl databases on the turkey (*Meleagris gallopavo*) and the mallard duck (*Anas platyrhynchos*) genomes. Expression of bird *CYP1C*s at the transcript or protein level has not been studied at all.

The objectives of this work were to define some features of *CYP1* genes in birds, particularly the *CYP1B*s and *CYP1C*s. We cloned cDNAs of quail *CYP1B1*, chicken *CYP1C1*, and quail *AHR1*, and determined basal mRNA expression profiles of the full complement of *CYP1s* and *AHR1* in chicken and quail embryos. Induction of *CYP1*s was studied in early embryos and yolk sac membranes after *in ovo* exposure to PCB126. We also compared syntenies around *CYP1B* and *CYP1C* genes in birds to those in other vertebrate species. The results indicate remarkable conservation of some features of *CYP1* genes among vertebrates, although differences were also found among birds, and between birds and other vertebrates.

## Results

### Cloning and sequence comparisons

**Figure 1:**
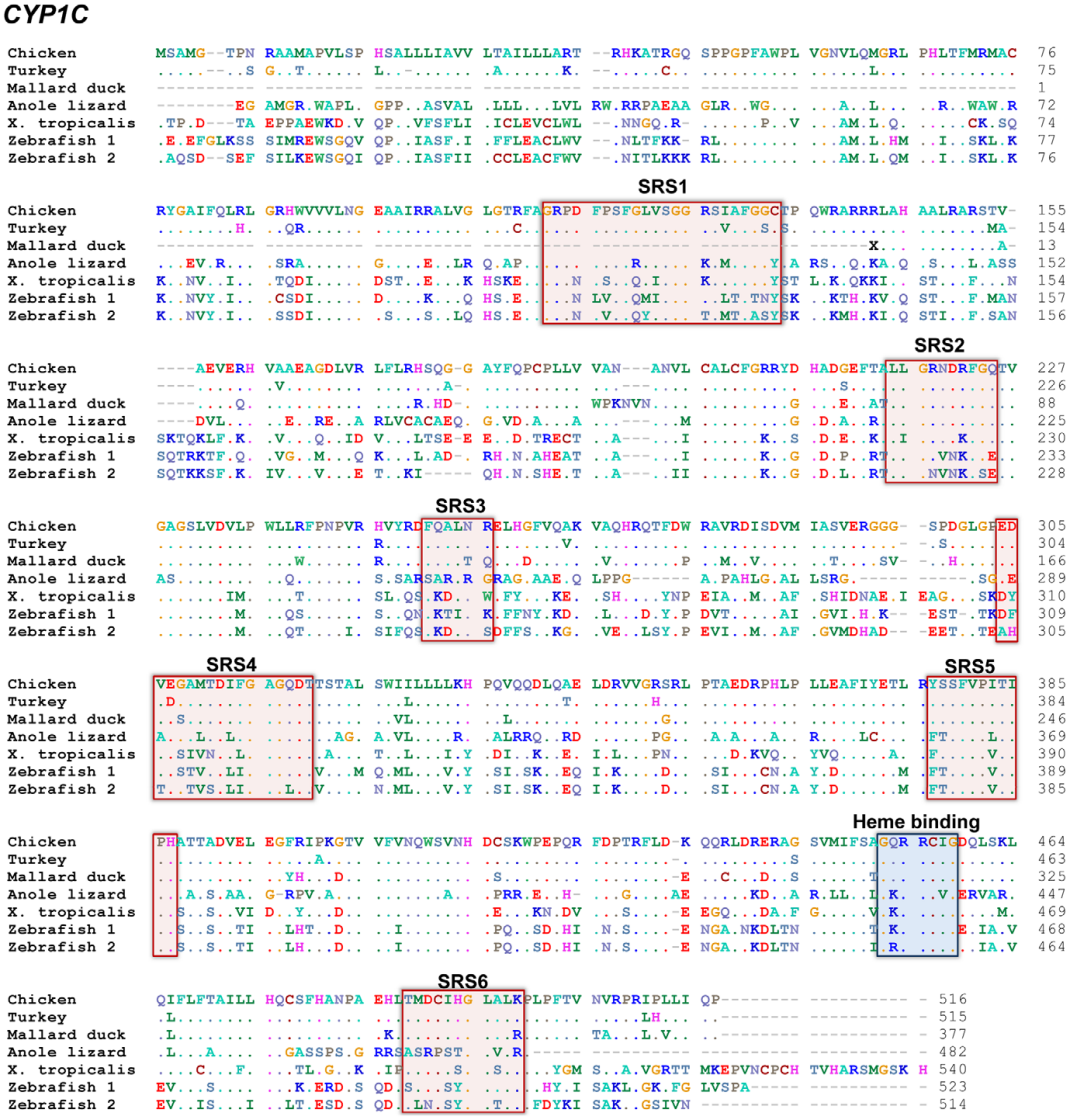
Cloned chicken CYP1C1 deduced amino acid sequence aligned with orthologous proteins in other species. Accession numbers are shown in Table 1.

Using primers targeting the predicted chicken *CYP1C1* [25] we cloned and determined the sequence of a cDNA covering the full coding region (1637 bp) of the transcript (GenBank: JN656933). The cloned chicken *CYP1C1* nucleotide and deduced amino acid sequences showed 99.6% and 99.0% sequence identity to the predicted transcript and protein, respectively. Figure 1 shows the deduced amino acid sequence of chicken CYP1C1 aligned with *X. tropicalis* CYP1C1, and zebrafish (*Danio rerio*) CYP1C1 and CYP1C2 [7]. CYP1C-like sequences found in the genomes of the turkey, mallard duck, and anole lizard (*Anolis carolinensis*) are also included in Fig. 1. Chicken CYP1C1 showed 93% and 87% amino acid sequence identity with the corresponding regions of the turkey and mallard CYP1Cs, and the identity was higher in the substrate recognition site (SRS) regions (94% and 93% for turkey and mallard CYP1C). Compared with *X. tropicalis* CYP1C1, chicken CYP1C1 exhibited 57% and 68% sequence identity in the full length protein and SRS regions, respectively. The anole and chicken CYP1Cs showed 54% and 69% sequence identity in the full length protein and SRS regions, respectively. Zebrafish CYP1C1 and CYP1C2 showed only 51% and 47% identity with chicken CYP1C1 in the full protein whereas slightly higher identities were observed in the SRS regions (54% for CYP1C1 and 49% for CYP1C2).

Extensive cloning efforts did not uncover a *CYP1C* ortholog in quail. PCR was performed with combinations of 12 forward and 10 reverse primers targeting *CYP1C1* regions that are conserved between the chicken, turkey, and mallard duck. In the reactions we used quail cDNA from whole embryos and from tissues which have a high *CYP1C1* expression in zebrafish (eye, brain, and heart [7]), or genomic DNA from a 4-day-old whole quail embryo. Amplification of quail cDNA using the quantitative real-time RT-PCR primers designed for chicken *CYP1C1* did yield a product, but that product was part of *CYP1B1*.

**Figure 2:**
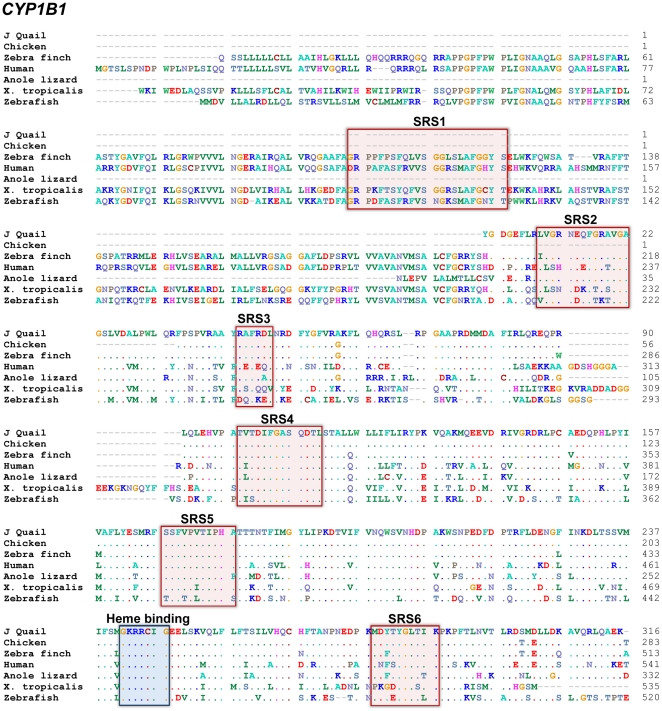
Cloned Japanese quail CYP1B1 deduced amino acid sequence aligned with orthologous proteins in other species. Accession numbers are shown in Table 1.

A cDNA resembling *CYP1B1* was cloned from quail (GenBank: JN656934), and a sequence with close similarity to *CYP1B1* was identified also in the zebra finch (*Taeniopygia guttata*) genome. The cloned quail *CYP1B1* sequence was 950 bp long, corresponding to approximately 60% of a complete coding *CYP1B1* sequence, and the predicted protein included SRS 2–6 (Fig. 2). The deduced amino acid sequence of quail CYP1B1 showed 99% identity with the corresponding region of the known chicken CYP1B1, and the SRS regions available in both predicted proteins (SRS 3–6) were identical. The quail CYP1B1 and the predicted zebra finch CYP1B1 showed 96% amino acid identity for the cloned segment and for the SRS regions. Quail CYP1B1 also showed 69%, 60%, and 58% sequence identity with same region of CYP1B1s in human, *X. tropicalis*, and zebrafish, and higher degrees of identity in the SRS regions (75%, 65%, and 69%, respectively).

**Figure 3:**
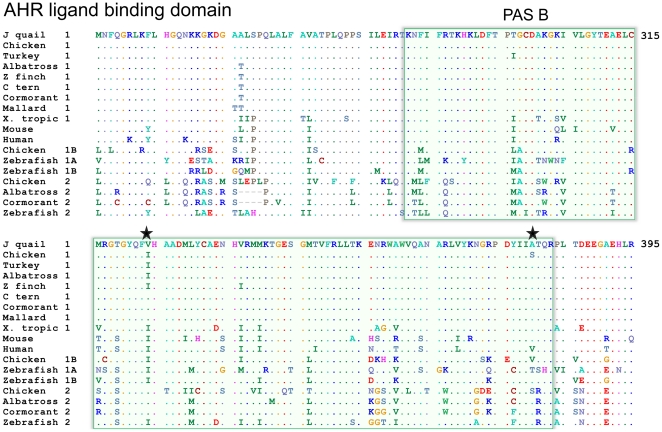
Amino acid sequence alignment of AHR ligand binding domains in Japanese quail and other species. In the figure “1”, “1A”, “1B”, and “2”, denote AHR1, AHR1A, AHR1B, and AHR2. Abbreviations: J quail = Japanese quail, C tern = common tern, Z finch = zebra finch, and X. tropic = *Xenopus tropicalis*. Accession numbers are shown in Table 1.

We also cloned a cDNA for quail *AHR1* (corresponding to amino acid numbers 231–395 of chicken AHR1), which includes most of the AHR ligand-binding domain (GenBank: JN656935). Figure 3 shows the translated cloned quail AHR1 sequence aligned with AHR proteins in seven birds, *X. tropicalis*, human, mouse, and zebrafish. Two clades of AHR proteins have been identified in fish and birds, the AHR1s and AHR2s. The quail AHR1 showed 99% sequence identity with AHR1 in other birds, while lower identities (62–72%) were obtained when compared to the AHR2 in chicken, albatross, and cormorant. Quail AHR1 showed 82% sequence identity to a third predicted AHR (AHR1B-like) protein found in the chicken genome (located next to *AHR2* on chromosome 7). The quail AHR1 sequence showed 70%, 84%, and 75% identity to the AHR1A, AHR1B, and AHR2 proteins in zebrafish, respectively. (Accession numbers of all *CYP1C1*, *CYP1B1*, and *AHR* genes mentioned here are shown in Table 1).

**Table 1:** GenBank or Ensembl accession numbers of the studied transcripts.

Species	Gene	Number
**Chicken**	***CYP1C1***	**JN656933 (cloned)**
**Turkey**	*CYP1C1*-like	ENSMGAG00000015774
**Mallad duck**	*CYP1C1*-like	ENSAPLG00000001387
**Anole lizard**	*CYP1C1*-like	ENSACAG00000013750
***Xenopus tropicalis***	*CYP1C1*	HQ018042
**Zebrafish**	*CYP1C1*	NM001020610
**Zebrafish**	*CYP1C2*	NM001114849
**Japanese quail**	***CYP1B1***	**JN656934 (cloned)**
**Chicken**	*CYP1B1*	XP419515
**Zebra finch**	*CYP1B1*-like	XP002191325
**Human**	*CYP1B1*	NP000095
**Anole lizard**	*CYP1B1*-like	XP003216002
***Xenopus tropicalis***	*CYP1B1*	HQ018041
**Japanese quail**	***AHR1***	HM053555, **JN656935 (cloned)**
**Chicken**	*AHR1*	AAF70373
**Turkey**	*AHR1*	XP003207170
**Albatross**	*AHR1*	BAC87795
**Zebra finch**	*AHR1*	XP002188964
**Common tern**	*AHR1*	AF192503
**Cormorant**	*AHR1*	BAD01477
**Mallard duck**	*AHR1*	AF192501
***Xenopus tropicalis***	*AHR1*	CX900378
**Mouse**	*AHR*	NM013464
**Human**	*AHR*	AAH70080
**Chicken**	*AHR1B*-like	ENSGALG00000004322
**Zebrafish**	*AHR1A*	NP571103
**Zebrafish**	*AHR1B*	AAY42958
**Chicken**	*AHR2*	XP421887
**Albatross**	*AHR2*	BAC87796
**Cormorant**	*AHR2*	BAF64245
**Zebrafish**	*AHR2*	CAK11168
**Japanese quail**	***EF1A***	**JN656936 (cloned)**

### Dioxin response elements

Putative dioxin response elements (DREs) were sought in the promoter regions of the chicken, turkey, and mallard *CYP1C* genes, using the sequence 5′-(T/G)NGCGTG-3′ [26], [27]. Within 10 kb upstream from the start codons of the *CYP1C* genes two putative DREs were found in chicken (at −458 and −1671 bp) and one was found in turkey (at −9193 bp). In the mallard duck genome database (version 1, Pre Ensembl) a fragment in the beginning of the *CYP1C* gene is unidentified (including approximately the first 140 nucleotides downstream from the start codon); putative DREs were found located at about 3600, 5500, and 5800 bp upstream from the 5′-edge of the unidentified region.

### Synteny

**Figure 4:**
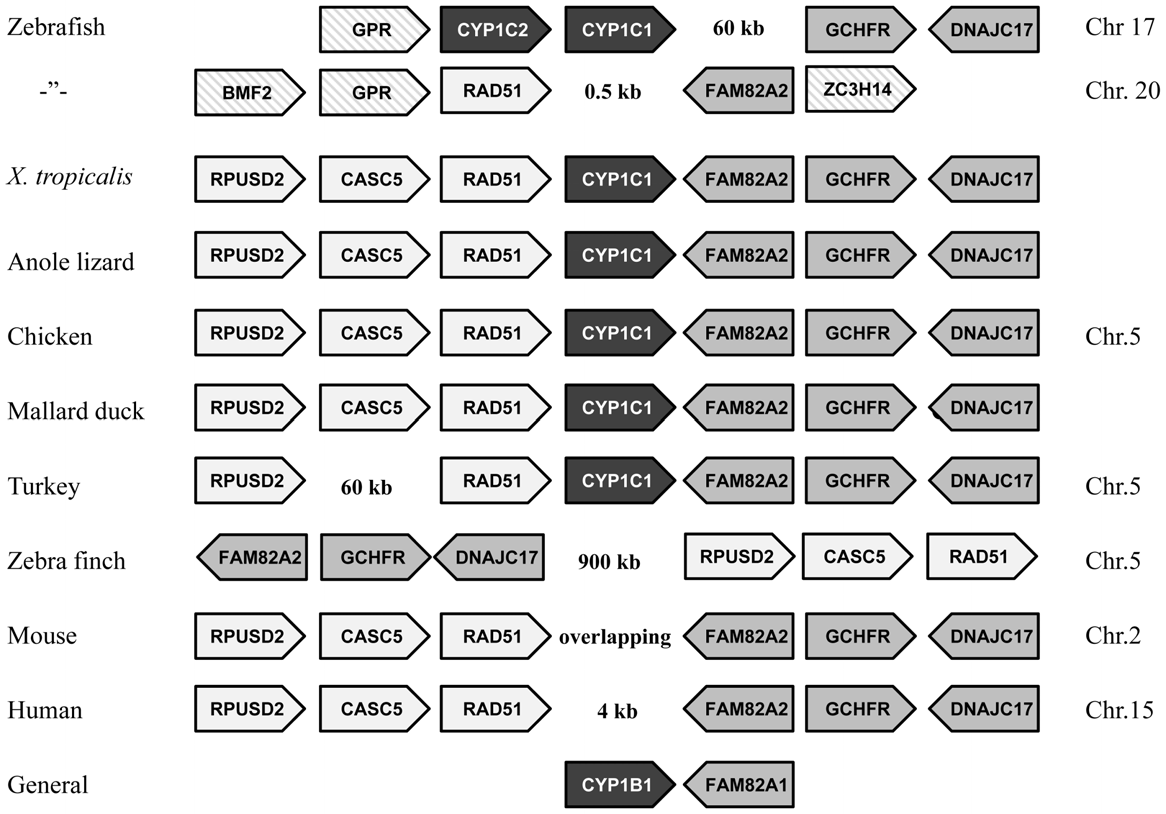
Synteny of *CYP1C* and *CYP1B1* regions in various species. Data were collected from the current assembly versions of the genome databases in Ensembl (http://www.ensembl.org/info/about/species.html): Zebrafish (version Zv9), *X. tropicalis* (version JGI 4.2, scaffolds: GL173137 and GL173263), anole lizard (version AnoCar2.0, scaffold GL343264.1), chicken (version 2.1, Chr. 5), mallard duck (version 1.0, scaffold 2370), turkey (version UMD2, Chr. 5), zebra finch (version taeGut3.2.4, Chr. 5), mouse (version NCBIM37, Chr. 2), and human (version GRCh37, Chr. 15). Zebrafish *CYP1C1* and *CYP1C2* have been mapped to chromosome 17 in previous zebrafish genome assemblies [7], but this mapping has not been confirmed in Zv9 as yet. The synteny of *CYP1B1* is shared by all species shown here. Chr = chromosome.

In order to examine the degree of conservation of the genomic region around the *CYP1C* locus we identified the three genes closest on either side of *CYP1C1* in chicken (*RPUSD2 - CASC5 -RAD51* - [*CYP1C1*] - *FAM82A2 - GCHFR - DNAJC17*), and localized the genomic position of orthologs to these genes in various species (Fig. 4). Our results indicate that all seven genes have the same order in *X. tropicalis*, anole, chicken, and mallard, while *CASC5* is absent in turkey (Fig. 4). Mouse and human have the same arrangement of these genes, except that *CYP1C1* is missing (Fig. 4). In the zebra finch genome, a segment including *RPUSD2*, *CASC5*, and *RAD51* was found located 900 kb downstream from *FAM82A2*, *GCHFR*, and *DNAJC17* on chromosome 5 (and *CYP1C1* was missing). In zebrafish, *RAD51* and *FAM82A2* were found next to each other on chromosome 20 whereas the two *CYP1C* paralogs are arranged in tandem on another chromosome [7] (the zebrafish *CYP1C*s were mapped to chromosome 17 in previous zebrafish genome assemblies, but this mapping has not been confirmed in Zv9 as yet). No shared synteny with chicken is found near upstream from the two zebrafish *CYP1C*s, but orthologs to *GCHFR* and *DNAJC17* are located downstream from the *CYP1C*s (Fig. 4). Zebrafish *RPUSD2* and *CASC5* were found on chromosomes 17 and 1, respectively. Three-spined stickleback showed a syntenic arrangement similar to that of zebrafish for these genes (in the stickleback *RPUSD2* and the *CYP1C*s are located on the same chromosome 3.3 Mb apart).

In all species examined here, *CYP1B1* was found adjacent to a *FAM82A2* paralog, *FAM82A1* (or *FAM82A*; Fig. 4).

In chicken and zebra finch, no *CYP1D1* ortholog was found in the region of *TMC1*, *ALDH1A1*, and *ANAX1*, which is the location for *CYP1D1* in rhesus monkey, zebrafish, anole lizard, *X. tropicalis*, and other species [12], [28]. Neither was *CYP1D1* found by blast searches in the chicken and zebra finch genomes.

### Tissue distribution patterns of *CYP1* and *AHR1* mRNA

**Figure 5:**
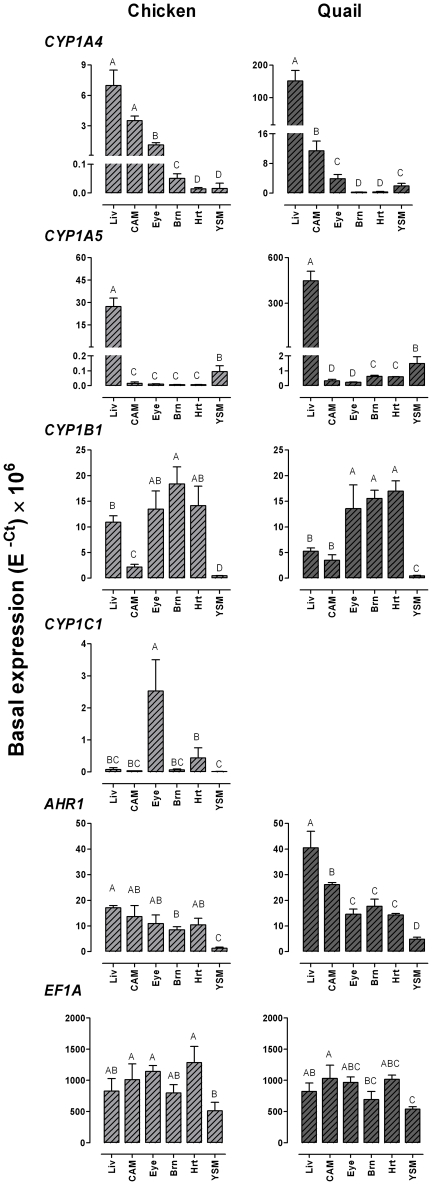
Tissue distribution of *CYP1* and *AHR1* mRNA in unexposed embryonic chicken and Japanese quail. Samples were collected at a similar developmental stage in chicken and quail (on incubation day 13 and 11, respectively). Levels of mRNA expression for *CYP1A4*, *CYP1A5*, *CYP1B1*, *AHR1*, and *EF1A* in chicken and Japanese quail, and for *CYP1C1* mRNA in chicken were determined by real-time RT-PCR. Results are shown as non-normalized data (E^−Ct^×10^6^; mean±SD). Statistical differences in transcript levels among tissues were determined by one-way ANOVA followed by Tukey's multiple comparisons test and are shown by different letters (*p*<0.05); *n* = 3–4 for chicken and *n* = 3 for quail. Abbreviations: Liv = liver, CAM = chorioallantoic membrane, Brn = brain, Hrt = heart, and YSM = yolk sac membrane.

Basal levels of *CYP1* and *AHR1* expression were determined in liver, chorioallantoic membrane (CAM), eye, brain, heart, and yolk sac membrane (YSM) in chicken and quail sampled on incubation day 13 and 11, respectively (equivalent developmental stages). Overall, the distribution patterns were very similar in the two species. In both chicken and quail, the liver showed the strongest expression of *CYP1A4* followed by CAM and eye (Fig. 5). *CYP1A5* was considerably more strongly expressed in liver than in other tissues and *CYP1B1* was strongly expressed in eye, brain, and heart in both species (Fig. 5). However, our results suggest the levels of *CYP1A4* and *CYP1A5* mRNA were much higher in quail than in chicken, whereas *CYP1B1* was expressed at roughly similar levels in the two species. The expression levels of *AHR1* mRNA were fairly similar in liver, CAM, eye, brain, and heart in chicken while a somewhat larger variation was observed for *AHR1* expression among these tissues in quail (Fig. 5). YSM showed the lowest *AHR1* expression in both species (Fig. 5). We also analyzed *CYP1C1* mRNA expression in chicken; the eye showed the highest level followed by heart while other organs showed relatively low levels (Fig. 5). The reference gene, elongation factor 1 alpha (*EF1A*), seemed to be expressed at a similar level in chicken and quail, and in both species the six different tissues showed only small variations in *EF1A* mRNA expression levels (Fig. 5).

**Figure 6:**
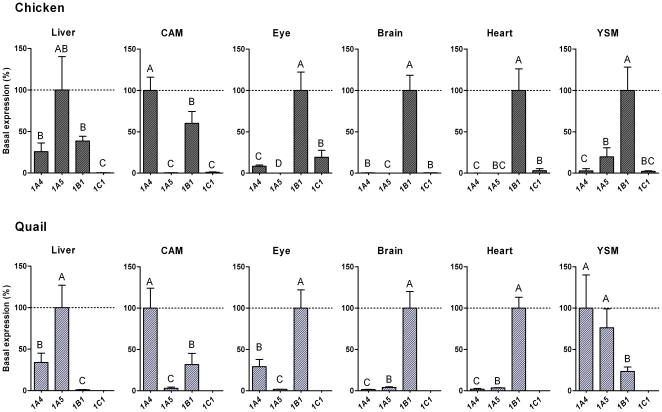
Expression patterns for *CYP1* mRNA in various tissues in unexposed embryonic chicken and Japanese quail. Samples were collected at a similar developmental stage in chicken and quail (on incubation day 13 and 11, respectively). Levels of mRNA expression for *CYP1A4*, *CYP1A5*, and *CYP1B1* in chicken and Japanese quail, and for *CYP1C1* mRNA in chicken were determined by real-time RT-PCR. Data were normalized (calculated by E^−ΔCt^) and results are shown as percentage of the gene with the highest level of expression within a tissue (mean±SD). Statistical differences among transcript levels within a tissue were determined by one-way ANOVA followed by Tukey's multiple comparisons test and are shown by different letters (*p*<0.05); *n* = 3–4 for chicken and *n* = 3 for quail.

The expression patterns of the various *CYP1*s within a tissue were compared in chicken and quail (Fig. 6). The results suggest that the *CYP1A*s and *CYP1B1* were expressed to a roughly similar level in chicken liver whereas the *CYP1A*s were much more strongly expressed than *CYP1B1* in quail liver (Fig. 6). In both species *CYP1A4* appeared to be the most strongly expressed and *CYP1A5* the most weakly expressed of the *CYP1*s in CAM, whereas *CYP1B1* seemed to be the dominant *CYP1* transcript in eye, brain, and heart (Fig. 6). *CYP1C1* was rather strongly expressed in the eye (in chicken). The expression patterns in YSM varied between the two species, *CYP1B1* looking more strongly expressed than the *CYP1A*s in chicken YSM, whereas the opposite was observed in quail.

### Expression patterns of *CYP1* and *AHR1* mRNA during early chicken embryo development

Expression of the four *CYP1s* and *AHR1* was determined in whole-body samples of unexposed chicken embryos collected on incubation days 1, 2, 3, 5, and 7 (Table 2). We found that all transcripts were expressed at all sampling time points. During the whole 7-day period studied a similar pattern appeared: *CYP1B1* was the most strongly expressed and *CYP1A5* was the second most strongly expressed of the *CYP1*s, while expression of *CYP1A4* and *CYP1C1* was considerably weaker than that of *CYP1B1* (Table 2).

**Table 2:** Relative levels of basal expression for *CYP1* and *AHR1* mRNA in early chicken embryos.

	Percentage of *CYP1B1* expression (mean ± SD)				
Transcript	Day 1	Day 2	Day 3	Day 5	Day 7
***CYP1A4***	0.4±0.5	0.2±0.0	0.1±0.1	0.1±0.1	0.9±0.1
***CYP1A5***	45±32	39±22	11±15	4±1	28±2
***CYP1B1***	**100±137**	**100±9**	**100±31**	**100±45**	**100±13**
***CYP1C1***	1.6±1.4	2.3±0.9	0.4±0.2	0.5±0.1	0.7±0.4
***AHR1***	51±56	158±86	81±30	24±7	57±4

Whole-body samples of unexposed chicken embryos were collected on developmental days 1, 2, 3, 5, and 7 and analyzed by real-time RT-PCR. Data were normalized (calculated by E^−ΔCt^) and results are shown as percentage of the *CYP1B1* mRNA level (*n* = 3). The embryos analyzed were staged according to Hamburger and Hamilton [63] and http://msucares.com/poultry/reproductions/poultry_chicks_embryo.html: Day-1 samples were taken after 31 h of incubation (stage 9, seven somites). At this stage the nervous system, eye, and heart have begun to develop. On day two (sampled at 50 h: stage 16, 19–22 somites) heart beats can be observed. On day three (sampled at 74 h: stages 20–21, 40–43 somites) nose, legs, and wings begin to appear. On day five (stage 27) the beak and reproductive organs start to form and sex differentiation occurs. On day seven (stage 31) feather papillae begin to appear.

**Figure 7:**
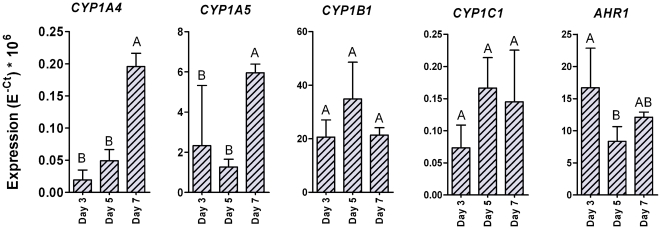
Basal levels of expression for *CYP1* and *AHR* mRNA during early development of chicken. Basal levels of mRNA expression were determined for *CYP1A4*, *CYP1A5*, *CYP1B1*, *CYP1C1*, and *AHR1* in whole-body samples of embryonic chicken collected on developmental days 3, 4, and 7. The samples were analyzed by real-time RT-PCR. Results are shown as non-normalized data (E^−Ct^×10^6^; mean±SD). Statistical differences in mRNA levels among development days were determined by one-way ANOVA followed by Tukey's multiple comparisons test and are shown by different letters (*p*<0.05), *n* = 3.

The time course of *CYP1* mRNA expression in whole-body samples from incubation day 3 to 7 is shown in Figure 7. The day 1 and day 2 embryos gave small total RNA yields, and less RNA was used in the assay for these samples than for older embryos. The embryos from the first two sampling times were therefore not included in the time course analysis. Within the study period *CYP1B1*, *CYP1C1*, and *AHR1* expression levels were relatively stable, while expression of *CYP1A4* and *CYP1A5* showed an increase from day 5 to day 7 (Fig. 7).

Table 3 shows the levels of *CYP1* and *AHR1* expression in whole-body, YSM, and CAM samples on day 7. While *CYP1B1* was the most strongly expressed of the *CYP1*s in the whole-body samples and CAM, *CYP1A5* showed the strongest expression in YSM (Table 3). In CAM the *AHR1* was more strongly expressed than the *CYP1*s.

**Table 3:** Basal levels of *CYP1* and *AHR1* mRNA expression in chicken embryos on developmental day 7.

	Expression (mean ± SD)		
Transcript	Body	YSM	CAM
***CYP1A4***	0.20±0.02	0.10±0.02	0.2±0.1
***CYP1A5***	6.0±0.4	**18±6**	0.11±0.04
***CYP1B1***	**21±3**	0.5±0.4	**2±2**
***CYP1C1***	0.1±0.1	0.04±0.03	0.2±0.1
***AHR1***	12±1	7±2	28±11

Samples of whole-body, yolk sac membrane (YSM) and chorioallantoic membrane (CAM) were collected from unexposed 7-day-old chicken embryos and analyzed by real-time RT-PCR (*n* = 3). Results are shown as non-normalized data calculated by E^−Ct^×10^6^. Each sample contained cDNA prepared from 30 ng of total RNA. The highest values among the *CYP1*s in a sample type are shown in bold.

### *CYP1* mRNA induction by PCB126

**Figure 8:**
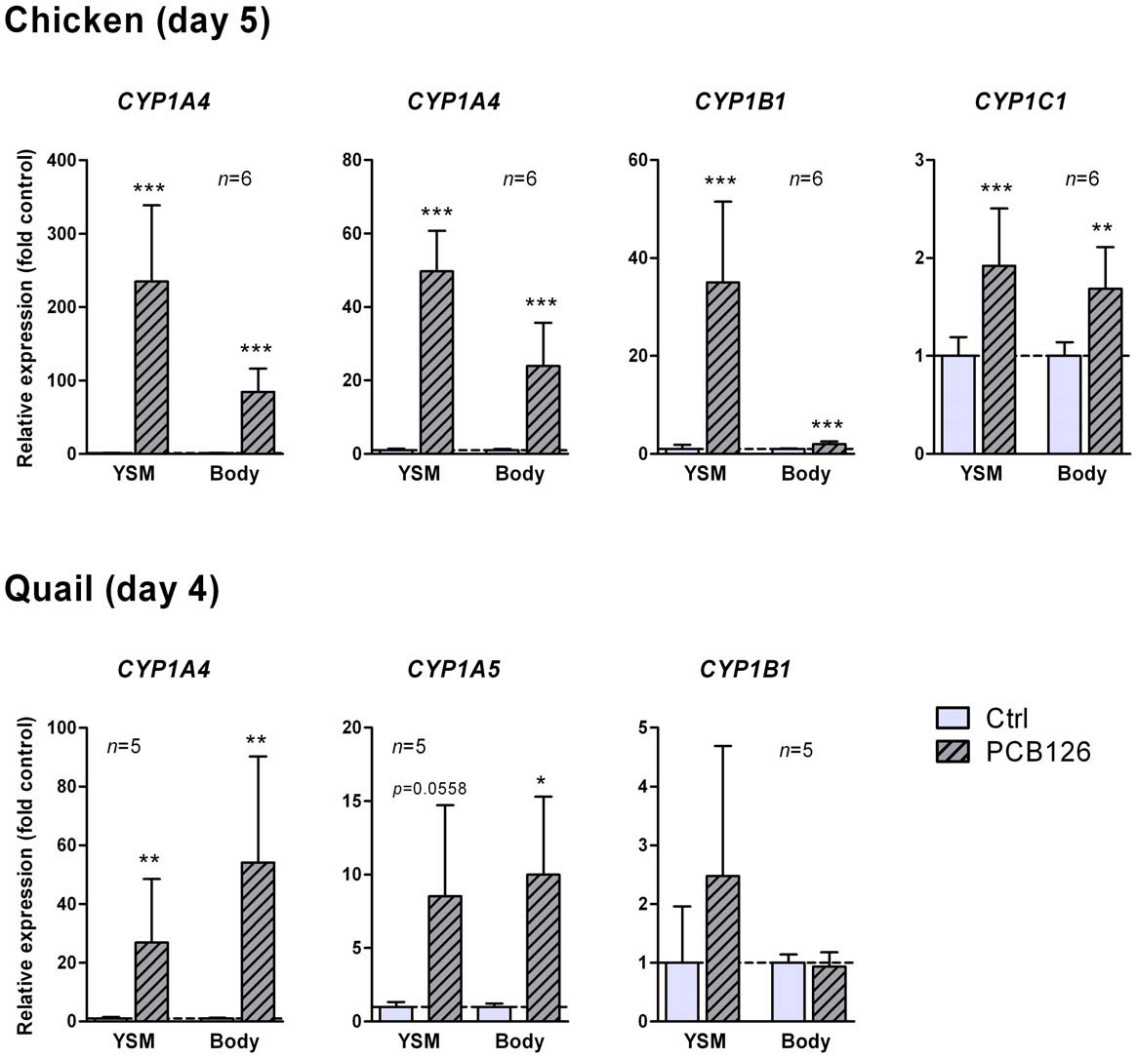
Effect of PCB126 on *CYP1* mRNA expression in chicken and Japanese quail embryos. Inducibility of the *CYP1*s was examined after exposure to PCB126 by egg injection on day 4 in chicken (*n* = 6) and on day 3 in quail (*n* = 5). Solutions of PCB126 dissolved in a peanut oil∶water emulsion were injected into the yolks, 0.2 µg PCB126 kg^−1^ to chicken and 200 µg PCB126 kg^−1^ to quail. Controls were injected with peanut oil∶water emulsion. After 24 hours of exposure yolk sac membrane (“YSM”) and whole-body (“Body”) samples were collected. The samples were analysed by real-time RT-PCR and relative expression levels determined by E^−ΔΔCt^. Statistically significant differences between the control- and PCB126-exposed groups were determined with Student's *t* test. Welch's correction was used when data did not show normal distribution. Significance levels are shown by asterisks *p*<0.05 (*), *p*<0.01 (**), and *p*<0.001 (***).

The effect of PCB126 on *CYP1* mRNA expression was determined in YSM and whole-body of chicken and quail embryos sampled 24 hours after injection. The control levels in whole-body and YSM were roughly similar in chicken and quail except for the level of *CYP1A4* in YSM, which was much higher in quail than in chicken. Chicken and quail eggs were injected with 0.2 and 200 µg PCB126 kg^−1^, respectively, doses that were shown by Brunström and Halldin [19] to induce hepatic EROD activity to a similar level in embryos of the two species. In chicken the PCB126 exposure induced expression of all four *CYP1*s in both YSM and whole-embryos (Fig. 8). Chicken *CYP1C1* was induced 2-fold compared with the control in both sample types, but the control level of this transcript was 10-fold higher in the whole-embryos than in the YSM. In quail, exposure to the 1000 times higher dose of PCB126 resulted in a significant induction of *CYP1A4* in both YSM and whole-embryos; *CYP1A5* showed induction in whole-embryos and a tendency for induction in YSM, whereas *CYP1B1* expression was not significantly affected by the PCB126 exposure in quail (Fig. 8).

## Discussion

**Figure 9:**
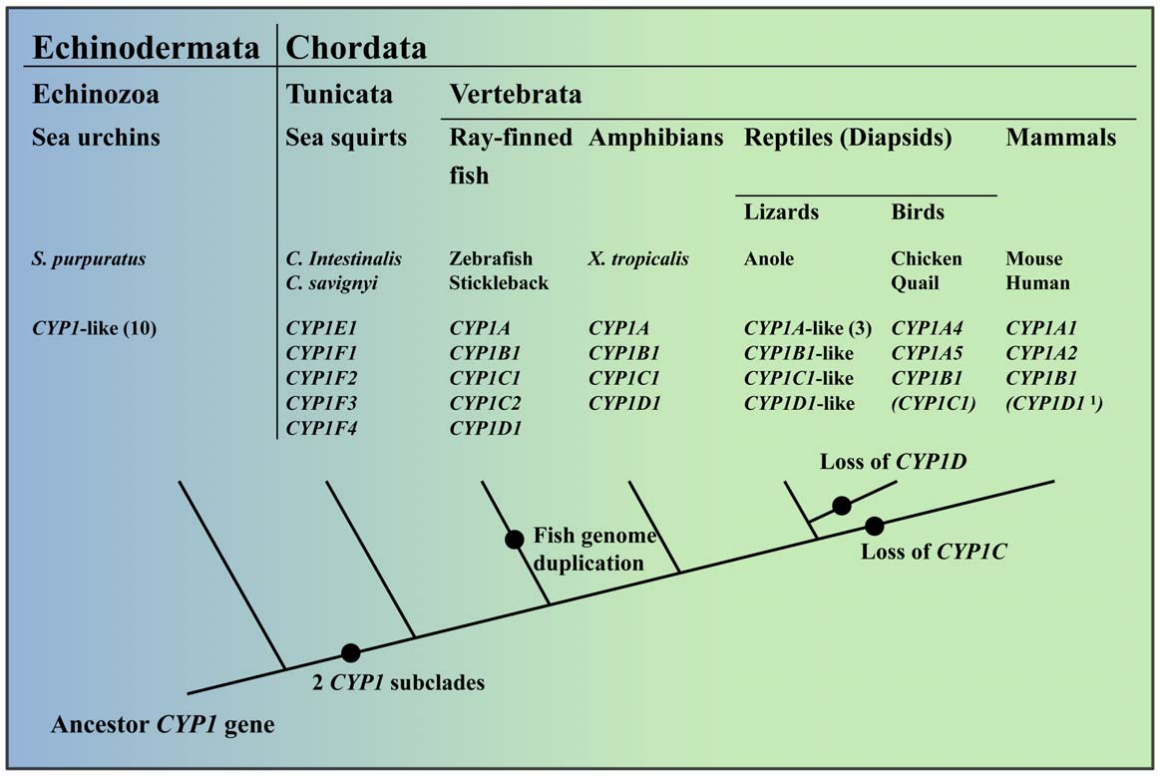
Evolutionary history of the *CYP1* family genes. The panel shows the presence of *CYP1*-like genes and *CYP1A*, *CYP1B*, *CYP1C*, *CYP1D*, *CYP1E* and *CYP1F* subfamily genes in various echinozoan, tunicate, and vertebrate classes. Suggested events of gene duplication and gene loss are shown by bullets. Ten *CYP1*-like genes have been identified in the genome of the sea urchin *S. purpuratus* and three *CYP1A*-like genes in the genome of the anole lizard. These genes have not been further studied. Genes within brackets indicate known or suspected absence in at least one species within the taxon (birds, mammals). Data were collected from Godard et al. [29], Goldstone et al. [25], Jönsson et al. [7], Goldstone et al. [13], Gao et al. [35], Jönsson et al. [6], and the anole lizard genome database (http://www.ensembl.org/Anolis_carolinensis/Info/Index). Footnote: ^1^ *CYP1D1* is a pseudogene in human, is expressed in macaques [12], [13], and appears to be absent in the mouse.

This study deals with *CYP1* genes and their expression in birds, focusing particularly on members of the *CYP1B* and *CYP1C* subfamilies. Phylogenetic analyses identify two major subclades in the vertebrate *CYP1* family, one comprising the *CYP1A*s and *CYP1D*s and the other comprising the *CYP1B*s and *CYP1C*s [13], [29]. Our results establish that the *CYP1B/1C* subclade, as well as the *CYP1A/1D* subclade, occurs in birds (although CYP1D genes seem to be missing). Genes related to the vertebrate *CYP1* genes have been found in the sea urchin *Strongylocentrotus purpuratus* (*CYP1*-like genes) and in the sea squirts *Ciona intestinalis* and *Ciona savignyi* (*CYP1E1* and *CYP1F1-CYP1F4*; Fig. 9), suggesting that *CYP1*-like genes were present in animals even before the Cambrian explosion [25], [30]. Goldstone et al. [25] found that the sea squirt genes can be assigned to either the *CYP1A/1D* subclade (*CYP1E1*) or the *CYP1B/1C* subclade (*CYP1F*s) whereas the sea urchin genes do not fall into either of these subclades. This suggests that the two *CYP1* subclades were established in early chordates (Fig. 9) [25].

The two subclades, multiple subfamilies, and several pairs of paralogs indicate that the *CYP1* gene family has undergone several duplication events. Duplication of whole genomes, genomic segments, or single genes are believed to be important for evolution of new functions [31]. Genes are also lost over time of evolution, supposedly as they become superfluous. *CYP1D1* is expressed in fish, *X. tropicalis*, and the cynomolgus monkey (*Macaca fascicularis*), while it is a pseudogene in human and some other mammals [6], [12], [13], [28]. Surprisingly, *CYP1D* has yet to be found in any bird species; whether it was lost early in the avian line is an important question. The *CYP1Cs* appear to have been lost in mammals generally [29].

Our findings hint that the role of *CYP1C*s is weakening also in birds. Chicken, quail, turkey, and mallard duck belong to the superorder Galloanserae (orders Galliformes and Anseriformes), while zebra finch belongs to the superorder Neoaves (order Passeriformes), which appears to have undergone a rapidly radiating evolution, comprising 95% of extant species [32]. Among the species studied, there was no clear evolutionary trend in the presence/absence of *CYP1C1*, i.e., it was found in chicken, turkey, and mallard, but not in their close relative quail. Furthermore, no hit for *CYP1C* was obtained in blast searches of the zebra finch genome database. Turkey *CYP1C* was predicted to be a pseudogene in Ensembl, having one small intron (5′-CCCC-3′). This could be an inaccurate prediction due to sequencing error since removal of one cytosine and use of the intron as a codon results in a translated protein sequence highly similar to the chicken and mallard CYP1Cs (Fig. 1). However, only one putative DRE was found within 10 kb upstream from turkey *CYP1C* (at about 9 kb upstream from the start codon), which raises a question about the inducibility of this gene via the AHR. The mallard *CYP1C* gene had three putative DREs in the upstream promoter region, but gene prediction is uncertain since part of the promoter sequence (and the start codon) was unidentified. In the zebra finch two *CYP*s that are not *CYP1C* orthologs were found at other places on chromosome 5. These genes were *CYP2R1* (vitamin D 25-hydroxylase) and *CYP46A1* (cholesterol 24-hydroxylase) and the regions around these *CYP*s exhibited a high degree of shared synteny in zebra finch, chicken, and turkey. In the zebra finch, genomic rearrangement appears to have occurred precisely at the location equivalent to that of *CYP1C1* in the other birds (Fig. 4). Whether this represents true genomic rearrangement rather than misassembly is not clear at present. That some *CYP1C1* roles may be diminishing in birds is suggested also by the relatively low basal level of *CYP1C1* mRNA expression in chicken embryos (except in the eye and heart) and that *CYP1C1* is only slightly inducible by PCB126 in birds, while *CYP1C1* is relatively strongly inducible in fish [7], [10], [33], [34], [35]. To resolve the evolutionary fate of *CYP1C1* in birds, this gene needs to be studied in a larger number of species.

The region around the *CYP1C* locus shows a high degree of shared synteny in many vertebrate species, including the anole lizard. The genes next to *CYP1C1*, *RAD51* and *FAM82A2*, code for highly conserved proteins with developmental functions, i.e., DNA repair (*RAD51*) and differentiation and apoptosis (*FAM82A2*). These two genes contain several putative DREs in their promoter regions (within about 2 kb upstream from the start site) in zebrafish, chicken, *X. tropicalis* and human. Interestingly, an ortholog of *FAM82A2*, *FAM82A1* (*FAM82A*) was found located next to *CYP1B1* on the chromosome in all species examined here (Fig. 4). This could mean that the regions of *CYP1B1* and *CYP1C1* derive from two copies generated by genome duplication in early vertebrates.

### Constitutive expression

The tissue distribution profiles for basal levels of *CYP1A*, *CYP1B*, and *CYP1C* mRNA in chicken and quail embryos (Fig. 5) were astonishingly similar to those in adult zebrafish. In both fish and birds there are distinct differences in mRNA expression patterns between the subclades, with *CYP1A* (and *CYP1D* in fish) being more highly expressed in the liver and *CYP1B*/*CYP1C* being more highly expressed in eye, heart, and brain [7], [13]. Expression of *CYP1A4*, *CYP1A5*, and *AHR1* has been determined also in the cormorant (*Phalacrocorax carbo*), and the mRNA expression profiles of these genes in liver, heart, and brain were almost identical to those in chicken and quail embryos [36]. The tissue distribution of the two bird *CYP1A*s also showed some similarity to the distribution of the two *CYP1A*s in mammals, in which *CYP1A1* (ortholog of *CYP1A4*) is widely expressed whereas *CYP1A2* (ortholog of *CYP1A5*) is expressed strongly only in the liver [37].

Regarding distribution of *CYP1B1* in mammals, heart and brain show high expression levels in mice, while in human *CYP1B1* is highly expressed in heart and weakly expressed in brain [38]. Unlike the quite strong *CYP1B1* expression in the bird embryo liver, *CYP1B1* is weakly expressed in the mammalian liver [38]. It is a curious finding that the eye is the tissue where *CYP1C1* was most highly expressed in the 13-day chicken embryo. Notably the two *CYP1C*s show a high expression also in the adult zebrafish eye [7]. The roles of CYP1C in the eye are not known. In mammals, CYP1B1 is critical for normal eye development [39], [40]. CYP1Cs might share this role in other vertebrates.

The relatively high level of *CYP1B1* expression over the course of development in most tissues (excluding liver) in both chicken and quail embryos suggests that it plays a role in developing birds. *CYP1B1* mRNA has been localized (by *in situ* hybridization) to the developing eye, neural system, somites, and other structures in chicken embryos [8]. In embryonic zebrafish the basal level of *CYP1B1* mRNA expression peaks at approximately 32 hours after fertilization, in a period of organogenesis and differentiation, while *CYP1A* expression peaks about three weeks after fertilization, and expression of the *CYP1C*s peaks just after hatching [33]. Choudhary et al. [41] found that in developing mice *CYP1B1* is expressed during neural patterning and somitogenesis, organogenesis, and later fetal stages, whereas *CYP1A1* is expressed during gastrulation only, while *CYP1A2* expression was not detected at all. Thus CYP1B1 appears to be important in early development in zebrafish, chickens, and mice.

Endogenous substrates for CYP1 enzymes include various eicosanoids, estradiol, retinoids, and uroporphyrinogen and melatonin (reviewed by [1], [4], [5]). Chambers et al. [8] found that CYP1B1 can catalyze a step in the formation of retinoic acid, and suggested it is involved in retinoid-mediated patterning. The CYP1s also have been suggested to metabolize endogenous AHR ligands that could play roles in development and differentiation [42]. One molecule which could have this function is the tryptophan photoproduct 6-formylindolo[3,2-b]carbazole (FICZ) [43]. FICZ activates the AHR at hormonal levels and is metabolized by human CYP1A1, CYP1A2, and CYP1B1 with an extraordinarily high efficiency [44]. In conclusion, the CYP1 enzyme functions may include synthesis and degradation of endogenous AHR agonists and other signaling molecules.

### The *AHR* and *CYP1* mRNA induction

Induction of *CYP1A*, *CYP1B*, and *CYP1C* genes and most toxic effects of TCDD are mediated via the AHR. The *AHR* genes are divided into two clades, *AHR1* and *AHR2*. Mammals have a single *AHR1* gene and no *AHR2* gene, while fish and birds have both *AHR1* and *AHR2* genes [36], [45]. In zebrafish, AHR-dependent toxicity and *CYP1* induction are mediated principally via AHR2, whereas in birds AHR1 seems to be prominent in these roles [27], [36], [45]. We found that *AHR1* mRNA was expressed at relatively high levels in a variety of tissues in both quail and chicken (Fig. 5). In the cormorant, *AHR1* mRNA expression shows a wider distribution and higher level than *AHR2* mRNA expression [36]. Features of the AHR may explain differences in sensitivity to dioxin-like compounds in certain mouse strains [46], and in birds, where for instance turkey and quail are much less sensitive than chicken [19], [47]. Frogs show a low sensitivity to dioxin toxicity and have an AHR with low dioxin affinity [48], [49]. The differing sensitivities to dioxin of mouse strains were shown to be due to differences in specific amino acid residues in the AHR ligand binding domain [46]. Similarly, differences in two amino acid positions in the ligand binding domain were shown to distinguish common tern (resistant) and chicken (susceptible) AHR1s [22]. The identity of the amino acids at these two positions predicted the sensitivity in a wide range of bird species [15]. Our results confirm those of Head and co-workers [15], showing that quail AHR1 has valine in position 324 and alanine in position 380 (the same as seen in the tern AHR1), while these positions have isoleucine and serine in chicken (Fig. 3). The turkey AHR1 has isoleucine and alanine in these positions, but also differs from the chicken and common tern AHR1 by having an isoleucine instead of threonine in position 297; the mallard AHR1 has two threonines instead of two alanines in the positions 256–257, but otherwise is identical to the quail AHR1 in the ligand binding domain (Fig. 3 and [15]). In addition to features associated with a resistant AHR, i.e., a low sensitivity to developmental toxicity of dioxins [50] and weak EROD response to PCB126 observed in embryo liver [19], quail exhibited a weaker *CYP1* mRNA induction by PCB126 than chicken despite the 1000 times higher dose given to quail.

The basal levels of hepatic *CYP1A4* and *CYP1A5* expression were much higher in quail embryos than in chicken embryos at similar stages, whereas basal *CYP1B1* expression looked largely similar in the two species. The significance of this difference between the genes is not understood. However, assuming there is a maximal capacity to synthesize the transcript for a given *CYP1*, the relative induction level would be low when compared to a high basal level. A higher basal level in the quail could explain the “weaker” induction of *CYP1A4* in YSM of quail than of chicken. Hence, a lower “fold-induction” level of a *CYP1* gene could reflect a high constitutive level of expression rather than a low responsiveness. However this would not explain the weaker induction of *CYP1A5* in quail vs. chicken, and the induction of *CYP1B1* in chicken but not in quail (Fig. 8). Rather these differences could be related to differences in AHR affinity for PCB126 not compensated for by the higher dose given to quail. In addition to AHR affinity of the inducer, the level of *CYP1* induction depends on the number of functional DREs in the gene promoters, epigenetic factors, interaction of the AHR with nuclear receptors (e.g., estrogen receptor), cofactors, the AHR repressor, etc. [26], [51], [52], [53], [54], [55]. Because the *CYP1*s are likely to be expressed in different cell types, it would be informative to study cell-specific induction of the *CYP1*s, and *CYP1C1* in particular, in chicken.

We previously found that the patterns of induction of *CYP1A*, *CYP1B*, and *CYP1C* were similar in zebrafish and *X. tropicalis* after exposure to PCB126 [6], [33]. In zebrafish embryos *CYP1A*, *1B1*, *1C1*, and *1C2* were induced 280-, 23-, 23-, and 40-fold versus the control and in tadpoles *CYP1A*, *1B1*, and *1C1* were induced up to 90-, 3-, and 8-fold versus the control [6], [33]. Together with the present results (Fig. 8) these findings indicate the *CYP1A* genes are more responsive to PCB126 than the *CYP1B*/*CYP1C* genes in developing animals. Thus, a strong induciblity appears to be an evolutionarily conserved feature of the *CYP1A*s, which could have to do with their functions.

### Conclusions

In this study we establish that *CYP1C1* is present in some birds. We show that *CYP1C1* mRNA is rather highly expressed in the chicken embryo eye. *CYP1B1* appears to have a high developmental expression in both chicken and quail. The similar distribution patterns of *CYP1C* and *CYP1B* transcripts in chicken and zebrafish imply that these *CYP1*s may serve similar functions in diverse vertebrates. Together with the absence of *CYP1C*s in mammals, the apparent absence of *CYP1C1* in quail, and weak expression and induction of *CYP1C1* mRNA in chicken suggests that *CYP1C*s have diminishing roles in tetrapods, which may be met by *CYP1B1*. Determining catalytic functions of CYP1 proteins in different species should indicate the evolving roles of these duplicated genes in physiological and toxicological processes. The studies reported here expand our view of the likely history and role of *CYP1*s.

## Methods

### Eggs

Fertilized eggs from chicken (White Leghorn) and Japanese quail were obtained from local Swedish breeders (OVA Production AB, Vittinge, and Olstorps Konservfabrik, Färgelanda, respectively). Eggs were incubated at 37.5°C and 60% relative humidity with automatic turning every 6 hours until sampled. The experiments of this study were approved by Uppsala Ethical Committee for Research on Animals (Uppsala district court; permit number C 282/9).

### Cloning and synteny

Complementary DNA of chicken *CYP1C1* was cloned using primers targeting the predicted full coding transcript. Total RNA was extracted from whole-body homogenate of chicken embryos (incubated for 5 days) using RNA STAT 60 (Tel. Test Inc. Friendswood, TX, USA). Subsequently mRNA was isolated from the total RNA using MicroPoly(A)Purist™ Kit (Ambion Inc., Austin, TX, USA), and cDNA was synthesized using the Omniscript reverse transcriptase kit with random hexamer primers (Eurofins MWG Operon, Huntsville, AL, USA). Amplification of cDNA was performed using the Advantage® 2 polymerase PCR kit according to instructions provided by the manufacturer (Clontech Laboratories Inc., Mountain View, CA, USA). The PCR products were resolved on a 1% agarose gel. A product of approximately 1600 bp was isolated and ligated into the pGEM-T Easy Vector (Promega, Madison, WI, USA), and the construct was transformed into *Escherichia coli* (TOP 10 Kit, Invitrogen, Carlsbad, CA, USA). Plasmids were purified from cultures of positive clones and sequenced (Eurofins MWG Operon). The sequences obtained were assembled using Sequencher® (Gene Codes Corporation, Ann Arbor, MI, USA), resulting in a consensus sequence corresponding to the full coding part of the predicted chicken *CYP1C1*.

We also cloned partial sequences of quail *CYP1B1*, *AHR1*, and *EF1A* using primers designed to target conserved regions of the chicken orthologs. Total RNA was prepared from whole-body homogenate of 4-day-old quail embryos using the Aurum™ total RNA fatty and fibrous tissue kit (Bio-Rad Laboratories Inc., Hercules CA, USA) and the RNA was reverse transcribed using the iScript cDNA synthesis kit (Bio-Rad). Quail cDNAs were amplified using the gene-specific primers with Advantage® 2 polymerase PCR kit (Clontech Laboratories Inc.). The PCR products were sequenced by Uppsala Genome Center (Rudbeck Laboratory, Uppsala) and sequences obtained were aligned using ClustalW in BioEdit [56]. The cloned sequences of chicken *CYP1C1* and quail *CYP1B1*, *AHR1*, and *EF1A* were assigned the following GenBank accession numbers: JN656933, JN656934, JN656935 and JN656936, respectively.

Seeking an ortholog for *CYP1C1* in quail we designed primers (12 forward and 10 reverse) targeting regions conserved between chicken *CYP1C1* and the *CYP1C1* predictions in turkey and mallard duck. In addition to cDNA from embryonic day 4 we used pooled cDNA from eye, brain, and heart (collected on embryonic day 11). A *CYP1C1* gene was also sought using genomic DNA isolated from quail whole-body homogenate (embryonic day 4) with DNeasy Blood & Tissue Kit (Qiagen). We also amplified quail cDNA using the quantitative real-time RT-PCR primers designed for chicken *CYP1C1* (Table 4), but the product obtained was of *CYP1B1*. Consequently, we did not find any ortholog for *CYP1C1* in quail neither in genomic DNA nor in cDNA made from total RNA.

**Table 4:** Sequences of all real-time RT-PCR primers used in the experiments.

Species/Transcript	GenBank Acc. No.	Forward primer (5′ to 3′)	Reverse primer (5′ to 3′)	Product size
**Chicken**				
***CYP1A4***	NM205147.1	ACTGCCAGGAGAAAAGGACAG	TCAAAGCCTGCCCCAAACAG	97
***CYP1A5***	NM205146.1	TTCACCATCCCGCACAGCA	GTTTCTCATCGTGATTCACTTGCC	109
***CYP1B1*** 1	XM419515.2	CATCTTCCTCATCAGGTATCCAAAAGT	GTACAGGAAAGCCACGATGTAG	130
***CYP1C1***	JN656933	TGTGCCCATCACCATTCCACAT	ACTGACCACTGGTTGACAAAGAC	99
***AHR1***	NM204118.1	GCTGTGATGCAAAAGGAAAGATTGTC	ATTCCACTCTCACCCGTCTTC	148
***EF1A***	NM204157.2	GATGTCTACAAAATTGGTGGCATTGG	GCTTCATGGTGCATCTCAACAG	140
**Japanese quail**				
***CYP1A4***	GQ906939.1	GCAAGTGAACCACGATGAGAAGAT	ACCACTTTGTCACCCTCTGTCC	111
***CYP1A5***	GQ906938.1	GCAAGTGAACCACGATGAGAAACT	TTTCCCCAATGCACCTCCTT	126
***CYP1B1*** 1	JN656934	CATCTTCCTCATCAGGTATCCAAAAGT	GTACAGGAAAGCCACGATGTAG	130
***AHR1***	JN656935	GCTGTGATGCAAAAGGAAAGATTGTC	CTCTCACCTGTCTTCATCATTCG	142
***EF1A***	JN656936	CTACAAAATTGGTGGCATTGGTACTG	TGACAACCATGCCTGGCTTCA	77

1 The same primer pair was used for chicken and quail *CYP1B1*.

The deduced amino acid sequences of the cloned cDNA were aligned with homologous sequences in other species and sequence identities were examined after pair-wise alignments using BioEdit. The SRS regions were localized out from Lewis et al. [57]. The synteny of *CYP1C* and *CYP1B* genes was determined in zebrafish, *X. tropicalis*, chicken, turkey, mallard duck, zebra finch, mouse, and human using the genome databases in Ensembl.

### CYP1 mRNA expression in chicken and quail embryos

Basal levels of *CYP1* mRNA were determined in unexposed chicken embryos sampled after various times of incubation (1, 2, 3, 5, and 7 days). Whole embryos and YSM were sampled separately (day 3, 5, and 7). In addition, CAM was collected from chicken at embryonic day 7. Finally, liver, CAM, eye, brain, heart, and YSM were collected from chicken and quail at embryonic day 13 and 11, respectively. All samples were frozen in liquid nitrogen and stored at −80°C.

Inducibility of the *CYP1*s was examined after exposure to PCB126 by egg injection. Injection solutions were prepared by dissolving PCB126 in a peanut oil∶lecithin mixture (10∶1, v∶w) which was emulsified in water (1∶1.5, v∶v) by ultra-sonication. Equivalent peanut oil∶lecithin∶water emulsions without PCB126 were prepared for controls. The emulsions were injected into the yolks of embryonated eggs after 3 (quail) and 4 (chicken) days of incubation. The volumes injected were 20 µl (quail) and 100 µl (chicken) corresponding to 200 µg PCB126/kg for quail and 0.2 µg PCB126/kg for chicken, doses that are high enough to induce hepatic EROD activity [19]. After injection, the holes in the shells were sealed with melted paraffin wax and the eggs were returned to the incubator. The embryos were sampled 24 hours later. Whole embryos and YSM were sampled separately, frozen in liquid nitrogen, and stored at −80°C.

### Quantitative real-time RT-PCR

Total RNA was isolated and DNase-treated using the Aurum™ total RNA fatty and fibrous tissue kit (Bio-Rad) according to Bio-Rad's instructions. The purity and quantity of RNA were determined spectrophotometrically (260/280 and 260/230 nm ratios were generally 2 or above) using a NanoDrop ND-1000 (NanoDrop Technologies, Wilmington, DE, USA). Total RNA was reverse transcribed using the iScript cDNA Synthesis kit (Bio-Rad).

Gene-specific quantitative real-time RT-PCR primers for chicken and quail *CYP1A4*, *CYP1A5*, *CYP1B1*, *AHR1*, and *EF1A*, and for chicken *CYP1C1*, were synthesized by Sigma-Aldrich (St. Louis, MO, USA) (Table 4). The predicted amplicon length was 75–150 bp. PCR was conducted using a Rotor Gene 6000 real-time PCR machine (Qiagen, Hilden, Germany). The 20-µl PCR reaction mixtures consisted of iQ SYBR Green Supermix (Bio-Rad), forward and reverse primers (5 pmoles of each; Table 4) and cDNA derived from 30 ng of total RNA. All samples were analyzed in duplicate with the following protocol: 95°C for 10 min followed by 30–40 cycles (cycle numbers varying with transcript levels) of 95°C for 15 s and 62°C for 45 s. At the end of each PCR run a melt curve analysis was performed in the range from 55°C to 95°C.

### Calculations and statistics

Finding a reference gene which is stable during development or which does not vary among tissues is difficult. Therefore, in some cases basal levels of *CYP1* and *AHR1* mRNA expression were calculated without normalization to an internal control (indicated in figure and table legends). In these calculations we used the equation E^−CT^ where E = PCR efficiency and CT = threshold cycle [58], [59]. The effect of PCB126 on mRNA expression was determined after calculation of E^−ΔΔCT^ [59]. *EF1A* was used as a reference gene for both quail and chicken; in neither of the two species *EF1A* was significantly affected by the PCB126 exposure. Mean values of E for within-experiment amplicon groups were determined by the LinRegPCR program using data within 10% of the group median [60], [61]. The E values obtained ranged from 1.83 to 1.92. Outliers were excluded based on the Grubbs test [62]. Statistical analyses were performed using Prism 5 by GraphPad Software Inc. (San Diego, CA, USA) with log-transformed data. The statistical methods used were Student's *t* test and one-way ANOVA followed by Tukey's or Dunnett's post hoc tests. Data were log-transformed before statistical analysis when the variances differed between groups. In the figures data are shown as mean+SD. Numbers of biological replicates used (*n*) are given in the figure legends.
